# The British Journals, &c.: Pathology, Practical Medicine, and Therapeutics

**Published:** 1842-10

**Authors:** 


### PATHOLOGY, PRACTICAL MEDICINE, AND THERAPEUTICS.

Rhubarb as an external Application in sloughing venereal Ulcers. The author reports one case in which rhubarb, applied in powder, was useful in severe and extensive venereal ulceration of the abdomen and wrist, after some other applications had failed. It caused, however, considerable pain and irritation, and could only be applied on alternate days. This was continued for six weeks, by which time the ulcers had "nearly healed."—Alfred Markwick, Esq., North Brixton; *Medical Gazette, July* 29, 1842.

---

Pathological Chemistry. From the chemical pathology of a case of scirrhous pylorus Dr. Bird infers: 1. That the gastric secretions are in this disease persistently acid. 2. That they are always more or less coloured, and contain, in suspension, sebacious particles. 3. That free hydrochloric acid exists, in considerable quantity, in the vomited fluids during the more irritative stage of the disease, and gradually disappears as the powers of life sink. 4. That free organic acids are secreted and are met with abundantly; at first co-existing with hydrochloric acid, and subsequently nearly replacing it. These acids are most probably lactic, acetic, and butyric.—Dr. Bird; *Medical Gazette, June* 10, 1842.

---

Ulcer on the Thorax communicating with the right Lung. The patient was a woman of thirty-one years of age, of scrofulous diathesis. During winter, when confined with a disease of the hip-joint, she had had occasional paroxysms of cough, with slight hæmoptysis; but in March she had regained flesh and strength, when, unfortunately, she caught fresh cold and had a return of cough, with profuse expectoration and perspiration. In May a scrofulous ulcer, which she had previously been troubled with, broke out on the right side of the thorax, and, assuming a phagedenic character, laid bare a portion of the fourth and fifth ribs. On the 25th of May the author's attention was directed by the patient to a curious noise proceeding from the ulcer, and which, on examination, proved to proceed from the expulsion and entrance of air in respiration; showing that a communication existed with the lungs. On the morning of the 24th of the following month she was found lying in her bed with such a profuse hemorrhage from the ulcer, that the person who saw it described it as "pumping out" each time that the patient breathed or attempted to speak. She died in ten minutes after. No examination of the body was allowed.—J. S. Allen, Esq., of the St. Marylebone Infirmary; *Lancet, July* 30, 1842.

---

Death from Peas. On the 27th of June a labourer, aged sixty, was brought to the hospital, who had been suffering since the 22d from an obstruction of the bowels, caused by a surfeit of gray peas on the 21st. When he came to the hospital he was in a sunken state, and died suddenly as he was being carried up in a chair to the ward. The rectum contained upwards of a pint of peas, which had been swallowed in a dry state, and almost without mastication, and had undergone no other change in their passage through the intestine than that of becoming swollen by the absorption of moisture They had formed a solid mass in the rectum, and filled almost the entire pelvis, pushing up the bladder (which was greatly distended) and prostate, so as to render the evacuation of urine, by any effort on the patient's part, impossible.—GEORGE JOHNSON, Esq., King's College Hospital; *Medical Gazette, July* 15, 1842.

---

PERFORATION OF THE STOMACH. The patient was forty-nine years of age, had been in indifferent health for some years. His appetite was, however, good, and he never suffered any inconvenience after eating beyond that arising from excessive flatulence. About six months before Dr. Kennion saw him, his symptoms had suffered an aggravation, in consequence of the death of a near relative; since which he had been subject to paroxysms of severe pain, generally coming on at night, and referred to the umbilical and hypogastric regions. Under judicious treatment his health was partially improved, but no radical amendment took place. At four o'clock, on the morning of June 12th, Dr. Kennion was called to see him, and found him labouring under all the usual symptoms of perforated stomach, with effusion into the peritoneal sac. The patient died at half-past nine.

On the anterior border of the lesser curvature were two irregular openings of about three quarters of an inch in diameter; the margins of which were as thin as a piece of paper. These perforations were at the distance of about an inch and a half from the pylorus, and were situated in the midst of a mass of cartilaginous substance, which involved the whole circumference of the stomach, three or four inches from the pylorus. — Dr. KENNION, of Harrowgate; *Medical Gazette, July* 1, 1842.

---

ON THE TREATMENT OF HEMORRHAGIC DIATHESIS. In almost all cases the blood which escapes from the incised or lacerated surface is preternaturally fluid. A deficiency of fibrin certainly exists, and this deficiency continues to augment in proportion as the bleeding continues. The number of the blood-corpuscles seems also to diminish. The coagulable power of that fluid is seriously impaired, and if a clot at all forms at the orifices of the torn or incised capillaries, it is loose, spongy, and easily detached; while a fresh clot is formed with more and more difficulty, if at all. Increased density of coagulum (which is what we want) is well known to depend on increase of the proportion of fibrin in the coagulating blood, as occurs in the inflammatory diathesis. But the proportion of fibrin is not to be here estimated in regard to the general mass of the blood merely, but rather in regard to the blood-globules; it being only when excessive in proportion to these, that the tendency to, and power of coagulation becomes most marked. Mere loss of blood produces a deficiency both of fibrin and globules, but not in an equal ratio. At first the latter are chiefly removed, and, consequently, at an early period of the case, loss of blood favours natural hæmoptysis, by increasing the proportion of fibrin to globules, and thereby augmenting the tendency to, and power of coagulation.

But the blood is not alone to blame. The capillaries and arterial tubes are deficient in contractility. In treating the disease, therefore, we must (as in the scrofulous diathesis, to which the hemorrhage bears a considerable resemblance,) prescribe a diet nutritious without being stimulating. Although the rapidity of many cases of hemorrhage preclude the possibility of any material benefit being derived from dietetic means, yet, seeing these are rationally indicated, it behoves us to avail ourselves of them.

The other means recommended by the author are acetate of lead and opium, given in heroic doses, and, if these disagree with the patient, sulphate of alum and potass, in doses of 15 or 20 grs.—nauseating remedies—and the sulphate of soda, which last he considers useful, by procuring serous discharges from the bowels, and thereby disposing the blood to "solid coagulation."

In regard to the relaxed condition of the capillaries, the author is of opinion that the acetate of lead will help to remove that state, while opium will calm the heart's action and the general circulation.

He is quite opposed to the actual cautery; and, as respects local treatment, would mainly rely on *pressure,* the bleeding part being first lightly touched with nitrate of silver. And, as "last, not least," in his list of remedies, he recommends *transfusion,* on the plain ground of the possibility of replacing, by this means blood "lamentably deficient in both globules and fibrin," with blood sufficiently abounding in both.—JAMES MILLER, Esq., Edinburgh; *Lon. and Ed. Journ. of Med. Science, July,* 1842.

---

INTERESTING CASE OF SYPHILIS. Mrs. B., a married woman and a mother, had an unhealthy-looking ulcer on the nipple, which, at one time was indolent, at another extended itself, and threatened to "excise" the nipple. An eruption subsequently appeared on the other breast and on the scapula, along with a large ulcer on each tonsil. The eruption consisted of many roundish spots, of the size of sixpences, of a dusky copper colour, constituting, as plainly as possible, that form of lepra described by Bateman as arising from venereal poison.

Some weeks before, a young woman had given birth to an illegitimate child, in the house of a mutual friend, Mrs. A. The woman either would not or could not suckle her infant, and Mrs. B. having at the time a child of her own at the breast, volunteered to give milk to the woman's infant, if brought to her house at particular hours. The child, a miserable creature, soon died; and it was after this that Mrs. B., as above described, presented signs of venereal infection. But Mrs. A., who, though married, had never borne children, and could, consequently, never have suckled the servant-woman's child, presented an ulcer of the nipple also, along with an eruption, differing, however, from that of Mrs. B., (which was decidedly leprous,) in being papular and covering the whole body.

On inquiry, it appeared that the infant of the woman had been covered with sores about the genitals and at every point. Yet the mother seems to have been healthy. It appeared also, that the child, when sleeping with Mrs. A., had been used to apply its lips to her nipple.

Subsequently to the affection of Mrs. B., her own infant had a leprous eruption on the posterior parts, exactly similar to that of the mother. The author concludes: 1. That a diseased infant may be borne by a mother *apparently* healthy; which must be admitted to be extremely possible. 2. That such an infant may, by sucking, communicate the disease to sound persons. 3. That the disease so communicated, may be followed by secondary symptoms. 4. That the disease may be different in such persons. That the disease imbibed may be communicated by suckling.—GEORGE LOWDELL, Esq., Lewes; *Medical Gazette, June* 17, 1842.

---

ON ALBUMINURIA. The author's opinion is, that the presence of albumen in the urine is produced by, and its proportional quantity is in a direct ratio to the degree of congestion of the capillaries of the kidney, from whatever cause that congestion may arise. Some experiments on rabbits are related, in which the renal vein was ligatured; and it would appear from these, that the secretion of urine of a highly albuminous character was the result of the operation referred to.—GEORGE ROBINSON, Esq., Newcastle-on-Tyne; *Medical Gazette, June* 3, 1842.

[In the third succeeding number (760) of the same journal Dr. Burridge, in a paper on "Granular Disease of the Kidney," remarks that the hyperemia produced by Mr. Robinson's ligature of the renal vein "does not constitute inflammatory action, being merely *one* of its elements;" and that "the absence of albumen from the urine cannot be admitted as a sound test of the absence of congestion."]

---

HEMORRHAGIC DIATHESIS. On the 18th of August, 1820, Dr. Allan was called to visit a boy, five years old, with a slight abrasion of the wrist, caused, a few hours before, by a bit of window-glass. A brother of the boy had died by hemorrhage from a wound equally slight. Both children were remarkably beautiful, with an unusual blush on the skin, auburn hair, and bright hazel eyes. Dr. Allan, along with Mr. Harvie, who had treated the first case, tried every means usual in such cases—such as alum, actual cautery, acetic acid, ol. terebinthinæ, bandaging, &c.—without the slightest good effect. On the evening of the 21st the patient appeared to have lost ground. On the 22d the action of the heart was indistinct and irregular; the urine, after becoming less in quantity and perfectly limpid, had ceased to be discharged during the last ten hours. On the 23d the breathing was slow, with frequent sighing; the action of the heart intermitting, the pulse imperceptible; but neither now nor heretofore any complaint of suffering on the patient's part. Slight oozing from the wrist on the 24th, and death in the morning of the 25th. Dr. Allan examined the wrist with a glass, and could only observe "a few small points, on which particles of colourless fluid rested." The author observes, "death appeared to take place more from decomposition of the blood—the serous portion seeming to drain off—than from loss of quantity; for the drops were slow, and many seconds between each."—Dr. ALLAN, of Haslar Hospital; *Lon. and Edin. Journ. of Med. Science, June*, 1842.

---

ABSCESS IN THE LUNGS, POINTING BELOW THE UMBILICUS. About ten months before his death, the patient, an intemperate working jeweller of thirty-four years of age, noticed a distinct, hard swelling under the short ribs on the left side. When Mr. Barker saw him, in October 1839, and about four months after its commencement, the tumour had a circumference about as great as that of "two hands opened and joined together," and extended on both sides of the mesial line, but principally to the left. There was distinct fluctuation in every part of the swelling. The patient complained of pain in the back and loins, particularly during night, with a heavy dragging pain between the shoulders. But there was no chillness or shivering; no pain in the chest; and no evening exacerbations. Some weeks after this, the tumour pointed and discharged itself. Hectic symptoms set in, and the patient died in the beginning of March.

On dividing the parietes of the abdomen, the muscles "were observed to be split up, as it were by a quantity of matter," not proceeding from the abdomen, but from the left lung which presented a cavity capable of containing ℥iv of pus. The matter had made its way through the external and internal oblique muscles of the abdomen, after passing through a small opening in the ninth intercostal space of the left side.—T. HERBERT BARKER, Esq., of Bedford; *Med. Gazette, July* 1, 1842.

---

FÆCAL VOMITING DURING THIRTY-FOUR DAYS. This is a rather uncommon case. The patient, a woman thirty-four years of age, was admitted into the hospital on the 3d of February, on which day she seems to have vomited fæcal matter, and continued to do so, with slight intermissions. Her bowels appear to have been constipated, until the 20th, and then to have been opened by enemeta. On the 12th her abdomen became covered with large, round vesicles, or rather pustules. She had always complained, from the time of admission, of pain in the epigastrium and abdomen, and had, in consequence, had leeches, bloodletting, and mercurials administered. On March 1st she appeared to be better, but on the 2d the vomiting returned, and on the 9th the pustular eruption of the abdomen reappeared, and a large patch of the integuments below, and to the right of the umbilicus, became discoloured. On this day she was seized with intense pain and shivering, the abdomen became tympanitic, and she died on the 10th.

A rupture was found in the lower part of the jejunum, through which a small quantity of thin fæcal matter had escaped into the peritoneal cavity. The intestine, for about sixteen inches, was gangrenous. Immediately below the rupture, and occupying the whole caliber of the bowel, was a solid, fleshy substance, about three inches in length, growing from the mucous membrane by a very narrow pedicle. This growth, by dragging down a portion of the intestine, had produced intus-susception.—J. S. ALLAN, Esq., of the Marylebone Infirmary; *Lancet, June* 4, 1842.

POST-MORTEM APPEARANCES OF A CASE OF GRINDERS' ASTHMA. The grinders are extremely averse to their bodies being opened, and take great care not to be overtaken with death in hospital. It is consequently rare that an opportunity occurs of examining the necroscopic appearances of consumption caused by inhaling dust and metallic particles.

In the present case—that of a man forty-nine years of age—the pleura adhered extensively on the left side, and was of a dark colour; both sides were studded with tubercles, of the usual light hue, most numerously along the anterior margins; there were two large cavities near the apex of each lung; the lining membrane of the bronchial tubes was natural, nor were there any tubercles around any part of these tubes. The principal peculiarity consisted in the great enlargement of the bronchial glands, one or two of which were of the size of walnuts, and contained a considerable quantity of a stone-like material, as hard as a piece of common lime-stone, but quite black like the glands themselves, which were as hard as fibro-cartilage. This induration was not confined to the conglobate glands, situated about the bifurcation of the trachea, but extended to the smaller ones under the pleura, some of which were of the size of a horse-bean. From microscopical and chemical examination, these concretions consisted simply of "animal tissue," and carbonate of line.—E. D. L. GILLAT, Esq. Sheffield; *Lancet, June* 18, 1842.

